# Of Epistemes and Insects: How *Drosophila* and Butterflies Shape Our Understanding of Radiation Risk

**DOI:** 10.1007/s10739-026-09851-0

**Published:** 2026-03-19

**Authors:** Donna M. Goldstein, Magdalena E. Stawkowski

**Affiliations:** 1 University of Colorado Boulder, Boulder, USA; 2 University of South Carolina, Columbia, USA

## Abstract

This paper explores the complexities of extrapolating insect data to understand nuclear exposure effects on humans. Within radiation research, animal studies are invaluable tools for understanding biological effects of radiation exposure. However, data are often employed selectively, exposing unsettled science in extrapolating animal data to human radiation effects. Here we focus on our understanding of the genetic effects of radiation exposure, and how the debates about the long-term effects on humans were molded by research carried out on the model organism known as *Drosophila*; disagreements among scientists occurred within the constraints of research on this model organism, creating what we see as a possible *Drosophila* bias. By tracing how *Drosophila* became the dominant model organism for radiation genetics, we show how this choice created an epistemic framework that often dismissed contradictory evidence from other organisms, including the more recent Lepidoptera data from Fukushima. Even when animal studies demonstrate harm, they are often dismissed due to the belief—and some of the science—of human exceptionalism: that humans are intrinsically more resilient than animals. In this work, we consider Michel Foucault’s ideas on epistemes as they apply to human exceptionalism in the context of radiation effects, mutation, and harm. By examining these two cases genealogically, we demonstrate how *Drosophila’s* dominance established frameworks of population resilience that persist to this day, leading to the dismissal of contradictory butterfly findings as inconsistent with “conventional understanding” rather than as challenges to what counts as legitimate evidence in radiation biology.

## Introduction

Animal and insect studies on the effects of nuclear exposure on humans has long been practiced but has also been a challenge. This challenge is particularly pronounced in fields such as toxicology, epidemiology, and radiobiology. Much of the research on the toxicity of substances and consumer product safety has hinged on establishing comparisons, links, and extrapolations from such animal and insect studies to human health outcomes. This process has been driven by the need to safeguard human well-being. Although there is consensus within the scientific community about the importance of animal studies in identifying potential human health risks, there are also debates about the limitations of extrapolating such data to humans. In the history of such efforts, phrases such as “in animal studies” or “in human studies” have become regular precursors to discussions of substantive characteristics. Nevertheless, the interpretation of data obtained from animal experiments has not been straightforward and has varied wildly due to the nature of substances and the animal in question.^1^

Because the study of radiation’s biological effects is conducted on animals, this is especially true of our understanding of the somatic and genetic effects of radiation exposure. The human-animal divide is perhaps best understood within a comparative and deeply historicized framework. In this essay, we critically examine the challenges of *extrapolation*, of applying findings from insect studies to the understanding of human genetic responses to nuclear radiation exposure. Insects have often been the focus of radiation research because of their sensitivity to environmental stressors and rapid life cycles, allowing scientists to observe physiological and intergenerational effects over a short period of time in a laboratory setting. In this essay, we focus on two distinct case studies that differ in location, that is where they took place (field, laboratory and country) and historical timeframe (one set of experiments in mid-20th century and the other more recently after the Fukushima nuclear accident). We employ Michel Foucault’s concept of the “episteme” because it summarizes our contention that some thoughts and scientific understandings we have now would have been impossible to envision in earlier epistemes (Foucault 1994). Foucault argued that a particular historical milieu may either allow for, or completely discount, an idea that is normative in another historical milieu or episteme. The episteme concept can help to shed light on the complexities of translating findings from insect research to humans in their appropriate historical contexts and help our inquiry about the animal-human line that informs and shapes our current understanding. These case studies encompass experiments on *Drosophila* spp. that investigated genetic mutations in populations in the 1940s, as well as more recent studies on mutant butterflies exposed to radiation after the nuclear accident in Fukushima, Japan.^2^

We advance two additional arguments. First, we highlight the profound scientific uncertainty and complexity inherent in radiation research on two model insects, resulting in the difficult and imprecise extrapolation of data from animals to humans. Second, we explore the historical and political context of nuclear research, especially during the Cold War era, when scientific uncertainty was amplified and polarized. As historian of science Angela Creager argued in *Life Atomic: A History of Radioisotopes in Science and Medicine*, Cold War scientific infrastructures shaped the priorities of biological research (Creager 2013). This context, much like other scientific research during this period, embodied the era’s tensions and attitudes toward the necessity of animal research. Finally, we suggest that there may be a “*Drosophila* bias” embedded in, and influencing, our understanding of the genetic effects of radiation research that was initiated during this important Cold War period. As Creager argues, model organisms are not simply passive substitutes for humans but deeply shape what questions are asked, what data are prioritized, and what biological effects are imagined (Creager 2022). This early research and focus on *Drosophila* allowed for certain interpretations that may well have been dislodged if a different model organism had been chosen and used with the experimental apparatus of the field and laboratory designed experiments of the time. Our comparative approach examines radiation genetics across two distinct epistemes: the mid-20th century framework in which *Drosophila’s* resilience and population recovery became the template for our understanding of radiation effects, and the post-Fukushima moment when butterfly data revealed cumulative genetic damage challenging this framework. We ask not “what if butterflies had been chosen instead?” but rather, how does the historical dominance of *Drosophila* as a model organism shape and constrain what counts as legitimate evidence of radiation harm? This paper is thus not a counterfactual analysis but a genealogical analysis of how the choice of organisms structures and constrains scientific knowledge and its limits. We have seen that when animal studies implicate harm to humans, they are frequently diminished based on the belief in human exceptionalism, the idea asserting that humans are inherently different or more resilient than animals. We are familiar with this manner of thinking; it is coincident with Cold War principles and with earlier epistemes. Our contention is that *Drosophila* was the model organism for understanding a range of questions in genetics and in radiation biology in the early 20th century, and the experiments and interpretations using this model organism may have biased the research on radiation genetics. The key scientists working on human genetics and radiobiology were originally trained in *Drosophila* genetics, many of them in T.H. Morgan’s laboratory at Columbia University. As Robert Kohler notes in his pathbreaking study of *Drosophila* as model organism (*D. melanogaster *in particular), “[t]he more elaborate the machinery of experimental production built around a species the more costly it would be to replace it with another species” (Kohler 1994, p. 51). He also added that *Drosophila melanogaster’s* character—vigorous, fertile, prolific, easy to manipulate, tolerant, and hardy—helped it become the standard instrument of experimental genetics and ultimately compatible with the material culture and experimental life of the scientists as well (Kohler 1994, p. 90). *Drosophila* was so well-known and successful as a model organism to genetics research that it inspired research on other organismic systems. Plant geneticist E.B. Babcock, for example, worked with the weedy genus *Crepis* (the hawkbeard plant), which became known as the “plant *Drosophila*,” seeking to replicate the phenomena of the “model organism” approach to plants (Smocovitis 2009).

In the long arc of scientific research on radiation effects since the early 20th century, we have noticed the selective referencing of outdated and somewhat problematic scientific research studies, particularly those conducted by the Atomic Bomb Casualty Commission (ABCC) and later the Radiation Effects Research Foundation (RERF) in Hiroshima and Nagasaki, Japan. These early studies mark the intersection of *Drosophila* genetics and human genetics. In one sense, this might be understood today as a perpetuated “radiation effects denial” among humans, or at least a seeming unwillingness to consider harms taking place in the present. The use of *Drosophila* as the model organism of the time, and the central place it took in evolutionary biology, could have influenced how mutations were conceptualized. It seems as if the model organism and the findings related to it were compatible with the epistemic principles of population biology that understood humans as far more resilient creatures. It seems to us that animal studies have historically been used to support claims that mutations are part of natural variation (which we see, as associated with Theodosius Dobzhansky’s perspective) and that radiation effects are manageable within frameworks emphasizing population recovery. We wonder if some of this approach has been biased by an over-reliance on *Drosophila*—as well as *Drosophila’s* compatibility with the mid-century’s understanding of human resilience. Taking into account *Drosophila’s* epistemic dominance at the time, we then explore the recent findings of radiation effects among Lepidoptera in Fukushima, Japan to consider the revelatory possibilities of an alternative model organism and to consider whether the current episteme—post-Fukushima Japan—opens the potential for new understandings of radiation effects among humans.

## The Drosophilists of 1940s Brazil

This story unfolds in Brazil in the 1940s,^3^ where *Drosophila’s* significance as a gateway model organism allowed portions of the scientific community to apply their expertise in fly genetics to early population biology and, eventually, to human genetics. The Brazilian field site is relevant not only to *Drosophila*, but also to the changing understandings of human genetics that were in motion as many of the early *Drosophila* genetics scholars transitioned to human genetics. *Drosophila* experiments in the field helped extend the reach of this tiny fly as a model organism. It is not by chance that many of the evolutionary and population biologists— or the “Drosophilists,” as they were called— of the 1920s to 1940s moved to studying humans by the 1940s. But our interest in this story is this: What might we see in the early work of Drosophilists that informs still-enduring scientific knowledge around radiation biology in humans? Many of the early Drosophilists later served on commissions and in research institutions responsible for helping understand the effects of radiation on the human body (or if it was not these self-same researchers, then their students). Our contention is that something about *Drosophila* as the model organism might have influenced the kinds of genetic (and therefore biological) effects that became recognized, and we wonder what kinds of limitations might have become embedded in our understanding of radiation effects because of this choice.

For our purposes here, this story focuses mainly on the disagreements over radiation effects that resulted in a historic debate between H.J. Muller and T.G. Dobzhansky, both of whom worked on *Drosophila*, as well as the context of the Brazilian Drosophilists who studied with Dobzhansky and carried out collaborative projects with him between 1943 and 1960 in Brazil. Dobzhansky’s fourth major visit to Brazil in the early 1950s and his work in Angra dos Reis are of special interest. Muller and Dobzhansky of course were two world-renowned geneticists, colleagues in the modern synthesis who at times disagreed, but who nevertheless helped strengthen *Drosophila* as a model organism and were involved in numerous scientific debates, some with each other, that are relevant to this history. Especially important is the time Dobzhansky spent in Brazil, helping to establish *Drosophila* research at the University of São Paulo, to mentor and direct fellow researchers, and to test some of his theories about *Drosophila* population biology, genetics, and evolution in tropical environments. Many researchers in Brazil who worked with Dobzhansky eventually studied other organisms and, later, humans, at leading radiobiological research centers such as at Oak Ridge National Laboratory in Tennessee, and Cold Spring Harbor Laboratories in New York, where they became world experts in understanding radiation effects on humans.^4^ Indeed, both Dobzhansky and Muller lived long enough to have broad insights into low-dose ionizing radiation and its effects on humans, and both participated in critical scientific conferences and panels evaluating human radiation health.

It is fair to say that many researchers in the early 20th century used *Drosophila* genetics as a gateway to research in human genetics and that some of what they learned from *Drosophila* influenced how they understood human genetics at a time before the discovery of the structure of DNA. The history and knowledge about *Drosophila* forms part of the assemblage of understanding of the risk-potential caused by low-dose ionizing radiation, an area of scientific research that is still controversial. This history of *Drosophila* thus adds to the puzzle of radiation risk assessment.

## Dobzhansky in Brazil

The prominent Ukrainian American geneticist, Theodosius Dobzhansky (1900–1975), author of the foundational work of evolutionary genetics titled *Genetics and the Origin of the Species* (Dobzhansky 1937), traveled to Brazil on four extended visits between 1943 and 1960 with support from the Rockefeller Foundation. Dobzhansky’s charismatic personality was still legendary among the Brazilian scientists interviewed in 2012 and 2015 at the University of São Paulo.^5^ Dobzhansky released laboratory-irradiated flies into the wild as part of his methods to see population differences between laboratory flies raised in enclosed spaces or population boxes under extremely controlled conditions and wild flies that had more range to travel. This topic became a critical question during the 1940s, and Dobzhansky arrived in Brazil believing that naturally occurring lethal genes would be less harmful to fly populations than those induced by radiation because natural selection limited their impact, while radiation-induced mutations had unpredictable effects.

Dobzhansky was long interested in taking what he learned in T.H. Morgan’s famed “fly room” at Columbia University and testing the principles of Mendelian genetics in natural populations, whether by releasing laboratory-modified flies into the wild or by studying populations in the wild, an approach noted by historian William Provine (2001, 1986, 1994). Morgan’s iconic genetic experiments with *Drosophila melanogaster* had some senior biologists of the period calling Morgan’s mutant flies monsters (deJong-Lambert 2012). Dobzhansky is remembered for many important scientific contributions (even beyond evolutionary genetics) including integrating field and laboratory studies (Kohler 1994), and utilizing predictive mathematical models such as the Hardy-Weinberg Law (Andre et al. 1989, p. 64)^6^—a theory that predicts that a population will remain at genetic equilibrium if five conditions are met.^7^ Dobzhansky’s interest in successfully integrating field and laboratory studies came to fruition, as the Angra dos Reis islands offered a unique natural laboratory. Morgan’s laboratory helped make *Drosophila* a model organism, because of its small size, requiring minimum care and having only four pairs of chromosomes. Most importantly, fruit flies breed quickly (they can lay hundreds of eggs in a few days), have short life cycles (about two weeks), and share 75% of the genes that cause diseases in humans. These characteristics make it easy to observe mutation rates.

The Brazilian researchers who became Dobzhansky’s academic partners were establishing their scientific disciplines at the newly formed Faculty of Philosophy, Sciences and Linguistics at the University of São Paulo, and Dobzhansky’s presence, direction, and global reputation afforded them the kind of intellectual cultural capital they needed to expand and to formalize high quality scientific research in Brazil. Dobzhansky’s main collaborator during his early visits was André Dreyfus (1897–1952), a geneticist who was a foundational member of the University during that period. Dreyfus’ death in 1952 meant that the direction of the Dobzhansky project was taken over by Crodowaldo Pavan (1919–2009), a population biologist and geneticist who also worked on the population genetics of *Drosophila*, on the enlarged chromosomes observed in the salivary glands. By the 1960s, Pavan was working at Oak Ridge National Laboratory, where he studied the effects of radiation and virus infection on chromosomal morphology and cellular genetics of *Rhynchosciara*, another fly. Upon his return to Brazil, he focused his research on questions related to the biological control of agricultural pests. In 2012, Goldstein interviewed some of Pavan’s then elderly and mostly retired former students whose generation experienced some of the benefits of the now established and prestigious University of São Paulo, and who worked in the disciplines still developing during Dobzhansky’s collaboration: population biology, *Drosophila* genetics, and human genetics.

Recent historical literature on Dobzhansky’s research program in Brazil has been slightly more critical of the work he accomplished there. Those who worked with Dobzhansky closely recognized that he greatly expanded and aided the research programs he was involved in, such as the one in Brazil. There, even today, he is greatly appreciated for the serious research program he designed and the publications he inspired (Monte et al. 2020). Yet, his overbearing personality and excessively directive style in his research collaborations, seen from a more contemporary perspective, is now considered as problematic (Magalhães and Vilela 2014; Lewontin et al. 2001).^8^ Colleagues who were also connected to Dobzhansky during his stay in Brazil recognized—perhaps later—that he was best defined as a Darwinian evolutionist and population geneticist rather than as a classical geneticist. This difference may be a critical one, as Dobzhansky was focusing on the stability of populations over generations, paying particular attention to how populations evolve over the long term, rather than on individual genes and their expression, as is the case with classical genetics. Much of this research took place prior to our basic understanding of DNA, when genetics was still in its infancy. From a contemporary standpoint, Dobzhansky’s research in Brazil at that time can seem simplistic. Additionally, his training was not centered entirely in genetics but instead on the evolution of populations over time, making some of his research designs appear problematic. Indeed, there were problems in Dobzhansky’s research design and, as some noted, a certain lack of rigor particularly in the statistical analysis of the results (Monte et al. 2020), and this was at a time when statistical reasoning was becoming *de rigeur*. Accordingly, his determination to prove his own theories correct may have weakened his experimental designs and strained his relationships with fellow researchers.

An illuminating account of Theodosius Dobzhansky’s deep interest in conducting research in Brazil appears in his memoir, *The Roving Naturalist: Travel Letters of Theodosius Dobzhansky*, edited by Bentley Glass (1980), where he recalled that as he flew over Angra dos Reis, Brazil, and observed the many small, seemingly uninhabited islands below, he realized they would provide an ideal setting to study *Drosophila* in their natural environment. Dobzhansky saw that the clusters of small islands would constitute a kind of “natural” population box (an experimental tool that allows for isolation and observation of insects in the laboratory) that could be controlled experimentally while still being in nature, a common idea about the value of island isolates for scientific study more generally in this period between the wars.^9^ Ove Frydenberg (1928–1975), a Danish researcher with a background in statistics and philosophy of science, came to Brazil to participate in Dobzhansky’s project. However, he expressed serious objections to Dobzhansky’s experimental design, claiming it would not test the intended hypotheses. As the authors of this retrospective of Dobzhansky’s work in Brazil (1940–1950) noted:

During the experiments, Magalhães, who was at the time a junior researcher with Dobzhansky, noted that he would have to release flies that had been created (and irradiated) in the university laboratory at Angra dos Reis, which he assumed may have muddled the results.^10^ The issue, it seems, and one that the researchers only acknowledged decades later, was that one of the markers *already* existed in the natural population, something Dobzhansky had a difficult time admitting. Dobzhansky belonged to one of two schools trying to understand the genetic diversity of natural populations: the mutational model and the heterotic model (Provine 2001 [1971]).^11^ Dobzhansky’s Brazilian team eventually released fruit flies into the field that had been genetically modified by radiation. The team of researchers then observed mutation in fruit flies across several generations. Dobzhansky’s knowledge of *Drosophila* and its characteristics in the laboratory was combined in this work with his skills as a field biologist interested in natural populations.

One of the emerging themes of Dobzhansky’s work in Brazil at the time revolved around the role of “lethal genes,” that can cause the death of an organism, usually due to specific mutations. This topic became a critical question during the 1940s, especially since so much of the research was supported by the Atomic Energy Commission. Dobzhansky believed that naturally occurring lethal genes would be less harmful than those induced by radiation, and was committed to the ideas at this time— what we might call the episteme—involving mutation, that radiation-induced mutations were categorically distinct from naturally occurring lethal genes because these newly introduced mutations could create more damage. This manner of thinking differed greatly from H.J. Muller (1890–1967) who had different thoughts about naturally occurring and radiation-induced genes.

H.J. Muller was a student of T.H. Morgan at Columbia University and later spent close to eight years in the Soviet Union, eventually becoming disillusioned with Soviet science during the Lysenko Affair and Stalin’s purges (Carlson 1981; Comfort 2012; Goldstein and Stawkowski 2015). Muller, importantly, had been the first to use X-rays to produce laboratory-generated mutations in *Drosophila* and his work examined the effects of these mutations on the genetics of the population. When he came back to the United States, he served on the commission to study the genetic impact of the atomic blasts on Hiroshima and Nagasaki survivors’ unborn children (along with many other colleagues called to this research endeavor at the time) and became known as a scientist who consistently warned about the negative effects of radiation on human genetics. Muller also won the Nobel Prize in 1946 for his work on radiation-induced gene mutations. During his Nobel Prize acceptance speech in 1946, he stated that mutations produced by irradiated genes are no more harmful than naturally occurring mutations, yet he still maintained a sharp concern about long-term radiation effects. This is important to our account as Dobzhansky and Muller served together on many of the important committees considering the effects of radiation on human populations. Muller remained concerned with, and became known for, his lifetime focus on the long-term deleterious effects of radiation while Dobzhansky understood mutations as part of a longer evolutionary process that resulted in variation. The historian of science John Beatty refers to this controversy of the 1950s and 1960s as the classical/balance controversy, with the classical position attributed to Muller and the balance view to Dobzhansky.^12^ While Beatty recognizes that both scholars were able to accommodate the findings of the other within their own position, he emphasizes that both were ultimately interested in social policy. As he wrote, “Dobzhansky and Muller not only hoped to understand human genetics and evolution in terms of *Drosophila* genetics and evolution, they also hoped to better the outcome of human evolution on the basis of their *Drosophila* research” (Beatty 1987, p. 302).

According to the geneticist James Crow, Muller, who remained a strong natural “selectionist” throughout his lifetime, was concerned about radiation effects.^13^ According to Crow, Dobzhansky, somewhat differently than Muller, favored a theory of “overdominance” that highlighted the importance of lethal genes, the same lethal genes that were central to, and confounding of, Dobzhansky’s *Drosophila* research in Brazil. Dobzhansky believed that since the lethal genes present in natural populations were subject to natural selection, they would be less deleterious than new genes introduced by exposure to radiation. So, a point of agreement is that both Muller and Dobzhansky were selectionists, but they differed on the extent of damage into the future that could be produced. However, the Brazilian researchers summarizing the findings of Dobzhansky’s Brazil work concluded their paper as follows:

These data clearly point out that in the laboratory there is no difference between the effects of irradiated lethal genes and natural lethal genes in hybrids of *Drosophila willistoni*. The experimental data therefore did not support the hypothesis that lethal genes newly introduced by radiation could produce more drastic effects than the ones produced by natural lethal genes in heterozygosis. (Pavan et al. 1958, p. 207)

This finding points to the possibility of Muller’s understanding being substantiated in Dobzhansky’s Brazilian research. It is also worth noting that while in Brazil, Dobzhansky was intent on showing that naturally occurring genes would be less deleterious than irradiated genes, but that the data did not bear out this hypothesis; Brazilian researchers eventually confirmed this finding later in their many publications. The idea that irradiated genes were no more deleterious than naturally occurring ones was anathema to Dobzhansky’s entire understanding of coadapted gene complexes. In his integrated system, any newly introduced mutation would have deleterious effects. As historians Monte Sião and Pereira Martins have noted:

Among the factors that could have contributed to the decrease in joint publications of Dobzhansky and the Brazilians we can also add the divergence between them concerning some scientific subjects, such as the effect of lethal genes. Beginning with Dobzhansky’s second visit (1948–1949) until the fourth one (1955–1956), this issue was under discussion. Some years later, a group of eight Brazilians published papers challenging Dobzhansky’s hypothesis. (Monte et al. 2020, p. 264)^14^

Despite the growing differences, Dobzhansky’s work and support for colleagues in Brazil helped establish the field of population genetics in Brazil, while also creating an important sphere of influence in the region and beyond.

As the geneticist James Crow wrote in 1987, by the 1950s both Dobzhansky and Muller found themselves at the center of questions related to radiation, genetics, and risk. Muller and Dobzhansky served together on the National Academy of Sciences Committee on the Biological Effects of Atomic Radiation (BEAR), which met several times during the mid-1950s and issued its report in 1956 (Crow 1987). According to Crow, the major disagreement on the committee was whether to use the Haldane-Muller genetic load argument to arrive at a risk estimate. He noted that many on the committee “did not want to measure the impact of mutation in terms of fitness: equating human welfare, now and in the future, to Darwinian fitness seemed simplified to the point of being misleading. Muller recognized this difficulty but could think of no other way to estimate the total impact, extending into the far future, from mutation at all loci” (Crow 1987, pp. 370–371). Eventually, the report contained a compromise that included an argument recognizing genetic load, but not exclusively. Dobzhansky and Muller could both sign on to the report, but some differences between them would persist, differences that Crow evaluated decades later, after extensive studies had been made in vertebrates, invertebrates, and plants, regarding average heterozygosities—the quality of having different alleles for a particular trait. Crow thought that neither Muller nor Dobzhansky foresaw the possibility of a great deal of neutral variation, that Dobzhansky’s ideas about overdominance did not hold up over time, and most significantly that:

Muller’s alarmist views of the hazards of radiation have prevailed rather than Dobzhansky’s more moderate views. In my opinion, Muller was *too* effective in cautioning against radiation risks, with the result that the public now has an irrational fear of low-level radiation relative to other risks. The fear, I suppose, has resulted more from the assumption of no threshold for carcinogenic effects than from the dread of genetic effects. In any event, the battle that Muller waged was certainly won: the present standards for radiation safety are more stringent than even he dared advocate. (Crow 1987, p. 379)

This assessment reveals the staying power of *Drosophila*-based understandings of radiation resilience through time. Crow’s terminology of “irrational fear” reflects the sort of epistemic frameworks we are untangling: broad-sweeping assumptions that populations can recover and that mutations are manageable. Importantly, contradictory claims are often labeled as “irrational” and therefore as merely psychological responses, not based on scientific evidence (Stawkowski 2017, 2025). Crow (1987) interpreted the classical–balance debate as a victory for Muller, whereas Beatty (2006) later argued that the apparent consensus among committee scientists may have obscured real disagreements, serving to preserve their collective social and political authority and limit lay interventions in determining the maximum acceptable dose of radiation. Beatty has argued that the report expressed consensus rather than disagreement “motivated by a genuine concern not to allow a less knowledgeable group to take advantage of the geneticists’ disagreements and impose far less justifiable standards” (Beatty 2006, p. 65). These debates about radiation hazards animated debates about radiation and genetics into the future.^15^ And were already to some extent an in-house debate among Drosophilists. Our interest here is in trying to imagine what might have been seen and debated if another model organism and its keeper(s) would have been able to enter this debate.^16^

We are not sure that—as Crow argued in 1987—Muller’s alarmist views of the hazards of radiation have fully prevailed over Dobzhansky’s more moderate views, but there is apparent continuity between what exactly was being looked at in *Drosophila*, even in the more questionable experiments, and the later hardening of positions about radiation effects. We posit that animal studies more generally, and here, insect studies, may have been used to generate a form of “radiation effects denial,” an outcome that some would contest. Many of the papers on insect radiobiology were published prior to 1975 and show that insects were, in general, less sensitive to radiation than vertebrates, and that mammals were more sensitive than birds or fish.^17^ The debates between Muller and Dobzhansky about lethal genes, natural selection, and radiation effects, based as they were on research on *Drosophila* genetics, established central assumptions about how populations respond to radiation exposure. Often, these assumptions emphasized recovery and resilience, a return to some sort of equilibrium in any population that experiences radiation effects. Indeed, Crow’s assessment that Muller’s “alarmist views” prevailed suggests to us that a bounded and constrained scientific consensus about radiation effects and dangers had emerged by the 1980s. Nevertheless, what we see repeatedly in the regulatory framework remains deeply rooted in assumptions about population recovery and resilience informed by *Drosophila* research. When butterfly research entered radiation biology decades later, it was within an epistemic framework already informed by *Drosophila* findings. Understanding this genealogy is key to making sense of why the case of the mutant butterflies in Fukushima has been so disturbing and why it continues to be dismissed by international regulatory organizations such as UNSCEAR as being inconsistent with conventional understandings of radiation science.

## Mutant Butterflies and Fukushima

While *Drosophila* spp. have been key to our understanding of genetic impacts of radiation and population dynamics in laboratory settings, Lepidoptera have provided novel insights into how environmental stressors, including radiation, affect their development and genetic stability across generations in wild habitats. Yet Lepidoptera research developed in the shadow of *Drosophila*, always measured against the resilience paradigm of the fruit fly. The Lepidoptera order, the second largest group of winged insects, surpassed only by beetles, is renowned not only for its incredible diversity, but also for its extensive use in scientific research around the world (Goldsmith and Marec 2010). Lepidoptera research complements the scientific work on *Drosophila*, by providing insights into species response in ecologically complex natural habitats rather than controlled laboratory settings. Their vividly colored wings, distinct life cycle stages, and rich biodiversity make them ideal subjects as model organisms to explore questions of adaptation, resilience, and mutation. For example, the moth *Bombyx mori*, used in silk production for over five millennia, became one of the first model organisms for studying inheritance, genetic variation, gene mapping, and the DNA repair mechanism, all of which are essential to understanding radiation-induced mutations (Goldsmith and Wilkins 1995). As modern scientific approaches developed, Lepidoptera research helped bridge the gap between laboratory studies on organisms like *Drosophila* and ecological studies on population recovery, genetic adaptation, ecological resilience, and eventually, the relationship between low-dose radiation exposure and health (Forister et al. 2010; Wagner 2020). From early work on mimicry and trait inheritance in the 19th century through studies of coevolution with flowering plants (Ehrlich and Raven 1964), Lepidoptera research developed its own distinct tradition, emphasizing questions of ecological interaction, adaptation, and biodiversity that complemented the genetic and chromosomal focus that dominated early *Drosophila* research. This central difference in how the two organisms were studied would shape how their radiation responses were interpreted.

While moths and butterflies have not become iconic in laboratory genetics in the manner that *Drosophila* has, they have nevertheless more recently become an important model organism in radiobiology and population genetics. With *Drosophila* research paving the way, the study of ionizing radiation’s impact on butterflies and moths began in the 1940s with the expanding use of nuclear technologies and the need to understand the biological effects of ionizing radiation on wildlife and ecosystems, and, by extension, the implications for human health. Some of the earliest studies on radiation effects on Lepidoptera included observations of butterflies and moths in the Marshall Islands where the United States conducted nuclear testing, including the largest thermonuclear device. These studies evaluated changes in insect populations through observation relative to the damage on natural vegetation by ionizing radiation from nuclear detonations (Armed Forces Special Weapons Project 1947; Cole 1951; Palumbo 1961; Woodwell 1963; Woodwell and Sparrow 1963).

Like *Drosophila*, Lepidoptera have large chromosomes which make it easier to observe genetic mutations and changes caused by various environmental pollutants, including radiation, across generations (Goldsmith and Marec 2010). The field of radioentomology—the study of the effects of ionizing radiation on insects—began after 1950, building on Muller’s *Drosophila* research from more than two decades earlier (Arthur et al. 2015). The radioentomological research on Lepidoptera primarily focused on their economic significance, including pest control, basic genetic research, and the use of radiation for removing pests from stored goods and agricultural commodities. These studies highlighted that radiosensitivity varies significantly among insect orders, with some species of Lepidoptera showing greater resistance to sterilization by ionizing radiation (sterilization doses range from 40 to 400 Gy). This resistance is linked to factors like developmental stage (pupae more sensitive than adults), sex (females being more radiosensitive than males), size (adults more radioresistant), and genetic difference (species adapted to diverse environments could have different radioresistance) which can reduce chromosomal damage and the likelihood of dominant lethal mutations (Ayres 1963, 1965; Carpenter et al. 2005). Radiosensitivity considers the end result measured—sterilization, death, or inability to reach the next stage of life.

It was only after the 1986 Chornobyl disaster, and later the Fukushima nuclear accident, that scientists became especially interested in what butterfly genetics might reveal about life in or near areas affected by nuclear fallout in assessing the impact of radiation on local ecosystems (Møller and Mousseau 2006; Mousseau and Møller 2014). Some butterflies and moths are particularly sensitive to changes in their habitats and to pollution, allowing scientists to monitor them as flagship species for assessing environmental damage and for conservation efforts (New et al. 1995). Anders Møller and Timothy Mousseau’s genetic studies of wild insect populations in Chornobyl, including butterflies, demonstrated significant effects from radioactive contamination (Møller and Mousseau 2006). Specifically, they documented increased phenotypic abnormalities, such as malformed wings, which diminished reproductive success in butterfly populations (Mousseau and Møller 2014). These effects contributed to significant population declines, showing how radiation disrupted genetic stability and fitness. Mousseau and Møller believe that the decline in Lepidoptera populations in Chornobyl may reflect the multigenerational accumulation of recessive deleterious mutations, further demonstrating the long-term genetic consequences of radiation exposure, findings that contradict the thesis that Lepidoptera are more radioresistant (Mousseau and Møller 2014).

But it is the Fukushima accident, and the mutant butterflies found there, that yet again spawned a heated debate about radiation effects. On March 11, 2011, the Fukushima Daiichi Nuclear Power Plant disaster marked the first major nuclear catastrophe since Chornobyl. Triggered by a 9.0 magnitude Tōhoku earthquake and subsequent forty-five-foot tsunami waves, several reactors at the plant were severely damaged. Three of these reactors experienced meltdowns, causing a release of toxic radioactive elements into the atmosphere. To prevent further explosions, the reactors were deliberately vented, dispersing radioactive particles. Government officials claim that health risks directly related to radiation exposure from Fukushima are low and that no clear *somatic effects*, such as cancer clusters, have been observed (WHO 2016). Much of the focus has been on the psychological stress caused by the evacuations and the resulting mental health consequences, which are cited across the globe as the primary public health impact of nuclear accidents, like that which happened in Fukushima (WHO 2016). At the same time, there remains virtually no acknowledgement of potential *genetic effects*, such as heritable mutations or congenital anomalies, that may only appear in future generations.^18^ The presence of butterfly mutations after the accidental release of radiation into the environment in Japan has thus become a highly contentious point, serving as both an environmental indicator of radiation effects and its hidden harms.

Shortly after, in August 2012, Joji Otaki, a professor at Ryukyu University in Okinawa, published a study (together with his colleagues) in *Nature’s* online journal *Scientific Reports*, titled “The Biological Impacts of the Fukushima Nuclear Accident on the Pale Grass Blue Butterfly.” The study revealed one of the more disturbing consequences of the Fukushima disaster: the emergence of mutant butterflies in the vicinity of the damaged nuclear plant (Hiyama et al. 2012). The butterflies not only showed phenotypic mutations, like malformed wings, broken antennae, and abnormal patterns on wings, but also physiological ones, genetic alterations that impacted butterfly body functions. The Japanese study forms part of a growing, yet contested body of scientific literature linking chronic low-level radiation exposure such as those released even from some normally functioning nuclear plants to intergenerational genetic damage, as was once proposed for atomic bomb sites.^19^ The research on butterflies not only showed inherited genetic damage in both the first and subsequent generations but also highlighted that these abnormalities and mutations persisted and even worsened in the offspring of exposed butterflies in the second and third generation. Specifically, these later generation offspring displayed a higher incidence of wing deformities, smaller body sizes, and other developmental issues compared to the first generation offspring, demonstrating that radiation-induced genetic damage could *accumulate* across multiple generations, intensifying impacts over time (Hiyama et al. 2012). Importantly, these findings underscore a paradox in how butterflies respond to radiation. While they are sensitive ecological indicators of environmental change, their radioresistance—the dose required to produce lethal effects like sterility—can be high. This does not mean they are unaffected by lower doses. In fact, sub-lethal effects, such as developmental abnormalities, cascade across generations and threaten survival and reproduction through cumulative genetic damage.

We might ask, at this point, why has it taken so long to recognize these abnormalities in Lepidoptera? Some possible explanations have been alluded to throughout this paper: the reliance on *Drosophila* as a model organism may have skewed our understanding of cumulative genetic damage. As we saw above, even Muller, who was involved in understanding radiation effects from an early stage in his career, was arguing that irradiated *Drosophila* and mutant flies in nature would not prove to have differential effects on populations. Dobzhansky, arguing instead that mutant flies in nature would be less deleterious than irradiated flies, also assumed that the genetic effects would be resolved by returns to equilibrium or co-adaptation. The idea of genetic damage—such as that found in Lepidoptera after the Fukushima accident—being transmissible, only became possible to speak about after Chornobyl and after Fukushima and in organisms that were not *Drosophila*.

The groundbreaking study of Fukushima butterflies was the first to systematically illustrate the multigenerational accumulation of genetic mutation in wild populations exposed to radiation. Prior research had often focused on single-generation effects or had found no effects at all (Goldstein and Stawkowski 2015). Although our understanding of butterfly recovery remains unclear, the scientific consensus is that insects are especially resistant to radiation, so this finding among Lepidoptera captured the imagination of many scientists interested in radiation effects in insects. According to the expert on radiation-induced genetic mutations in animals, biologist Timothy Mousseau, both naturally occurring and lethal mutant genes with negative effects are expected to diminish over time as carriers become less able to survive and reproduce.^20^ The speed of this purging process depends on factors such as the mutation’s fitness effect (how much the mutation interferes with the organism’s ability to survive), its dominance (how the mutation expresses its effects), and the effective population size (how many individuals contribute genetically to the next generation). Dominant mutations are removed quickly, while additive mutations take longer, creating a mutation-selection balance. Dominant mutations, which show effects with just a single copy of the mutated gene, are quickly discarded through the process of natural selection because their harmful impact is apparent. In contrast, additive mutations take longer to be eliminated because their effects depend on how many copies of the gene are present and how harmful they are.^21^ Recessive gene mutations can persist for generations, as seen in human diseases like Tay-Sachs and sickle cell disease, as they cause harm when two copies of the gene are inherited. Larger populations experience slower selection against mutations, particularly those with small effects. This process is part of what scientists describe as mutation-selection balance, where the introduction of new mutations is mitigated by their removal through selection.^22^ Despite differences in chromosome numbers, fruit flies and butterflies operate similarly in mutation-selection balance.

The Okinawa research team’s observation about mutant butterflies long after the initial accident at Fukushima contradicted common scientific knowledge about insect resistance to radiation effects. They found that the negative radiological effects seemed to manifest across generations, transmitted from parent to offspring (and intensifying in each generation). Their experiment exposed healthy butterflies to both low dose and higher doses of ionizing radiation in a controlled laboratory environment, comparing those collected near Fukushima to those from other regions of Japan. In May 2011, the researchers collected adult butterflies in Fukushima and surrounding regions. Two months prior, at the time of the disaster, these individuals were in the larval stage, surviving underground during winter months (Foote 2013). These butterflies (with an adult lifespan of one week), what we understand as the first generation, exhibited physical abnormalities such as deformed legs, broken antennae, and wrinkled wings at a rate 12% higher than the control group. The second and third generations saw mutation rates of 18% and then 34%, suggesting a direct correlation between genetic mutations and their inheritance. As time is one of the key factors in radiation research, along with dose, the study began to show more disconcerting revelations about butterflies as the months proceeded (and generations: F1, F2, F3, etc.). When the scientists collected 240 butterflies in September 2011, months after the accident, they discovered more pronounced and widespread abnormalities in 38.5% of the population. Since adult butterflies have a lifespan of only one week, it was evident that the mutations were not due to prolonged radiation exposure but were produced and intensified in the reproductive/generational process (Foote 2013). This indicates that radiation is causing mutations to the germline as well as somatic cells, with these abnormalities being inherited.

These were provocative results, and the study attracted considerable attention both inside and outside of Japan, some of it decidedly negative, with many criticizing the design and the conclusions of the study (Otaki and Taira 2018). The criticism, coming from the United Nations Scientific Committee on the Effects of Atomic Radiation (UNSCEAR 2014), echoed its earlier assessment of the Chornobyl disaster, declaring that radiation effects on “non-human biota” in highly contaminated areas were “unclear,” and “insignificant” in less contaminated areas. The findings expressed several lines of argument, but were all based on the same question: Can we really be sure the abnormalities were caused by radiation fallout from the Fukushima plant? Given the fact that so much of our understanding of radiation effects has been done in the context of *Drosophila*, it is not surprising that the butterfly research caused controversy (see Oskin 2012; Raines et al. 2020). The controversy surrounding the study highlights the complex and debated nature of interpreting radiation effects on insects, particularly in areas of different contamination levels. The mutant butterflies studied in this context reveal just how deeply our knowledge of radiation risk has been shaped by the historical dominance of the model organism *Drosophila*.

After Fukushima, the UNSCEAR published a report summarizing and evaluating research on the biological effects of the nuclear accident from their viewpoint, discussing the mutant butterfly study in detail (UNSCEAR 2014.^23^ The 2014 UNSCEAR report stated, for example, that:

[T]he relationship between exposure and effect has not been unequivocally established in these studies. Furthermore, the observations are not consistent with the Committee’s assessment and suggest that further analysis is needed to establish whether radiation exposure was an important factor, among many others, including the impact of the tsunami itself, in causing the environmental effects observed. (UNSCEAR 2014, p. 83)

The 2016 UNSCEAR maintains that these results are not entirely consistent with the conventional understanding of radiation biology. It is not compatible because the results, derived primarily from laboratory experiments involving external irradiation, don’t show any harmful biological effects associated with low-dose exposure. But if the radiation damage produces more lethal genes, as the butterfly study seems to suggest, then our understanding of radiation effects may be incomplete. Our understanding of lethal genes and radiation effects has been centered on the data derived from *Drosophila*, biasing how we understand all of this. The Fukushima study is currently challenging established frameworks in radiation biology and may require a reevaluation of the potential genetic impacts of radiation exposure on different species.

The Lepidoptera data seem to be telling us something new about insects, and, as with our understanding of other processes, might therefore offer some insight on humans as well. Thus far no peer-reviewed scientific study has definitively shown that humans experience similar outcomes as butterflies. The prevailing consensus among scientists is that humans are more resistant to radiation’s effects than butterflies (UNSCEAR 2014), a point consistent with some, but not all, of our understandings of radiation biology. *Drosophila*, for example, can be maximally irradiated and then bred and then come back to some stasis. What is happening to the population of butterflies in Fukushima has created an alternative possibility, one that remains uncertain. The controversy surrounding the Fukushima butterflies reveals just how deeply the *Drosophila*-informed episteme continues to structure what counts as credible and acceptable evidence in radiation biology. The UNSCEAR reports’ dismissal of butterfly findings as “not consistent with Committee’s assessment” demonstrates precisely what we are arguing in this paper: the choice of *Drosophila* as a dominant model organism is not neutral (UNSCEAR 2014, p. 83). In fact, as we have shown, it has created a framework in which evidence of cumulative, intergenerational, and intensifying genetic damage appears as an anomaly and not the norm. Our historical analysis shows that this conventional understanding was itself informed by specific organism choices, experimental design, and more importantly, the key scientists including Dobzhansky and Muller working within a Cold War context. The Fukushima butterflies are most likely not going to usher in a new episteme. Rather, they expose the constructed nature of the existing one, an episteme so deeply rooted in the fruit fly research that contradictory evidence is immediately dismissed rather than investigated.

The butterfly studies carry alarming implications for our understanding of radiation’s biological effects. They suggest that, despite challenges in extrapolating data from animals to humans, people might also be susceptible to transmitting damaged heritable germ-line DNA to future generations, a finding that the ABCC was reluctant to make (Goldstein and Stawkowski 2015). As Hiyama et al. note, “heritable germ-line genetic damage caused by low-dose exposure due to radioactive contamination in a species of butterfly has invaluable implications for the possible future effects of radiation on animals” (Hiyama et al. 2012, p. 8). Otaki warned it was too soon to jump to conclusions, saying his team’s results on Fukushima butterflies could not be directly applied to other species, including humans. Yet this caution is in tension with the *Drosophila* work discussed in the first half of this paper, where the transition from insect to human genetics occurred with great frequency, and the assemblage of knowledge learned in *Drosophila* work was brought to bear on our understanding of human radiation biology. Compared to *Drosophila*, the butterfly may indeed be more sensitive and alert us sooner to consider what the long-term effects of radiation exposure might be. It seems that both the population biologists and the UNSCEAR reports assume that the mutations will disappear over time and reach a new stasis, rather than accumulating as they do in these recent studies of butterflies in Fukushima.

## Conclusions

Several issues gain clarity in our examination of insects and the kinds of studies done with them in two distinct epistemes. In other approaches we have undertaken—both collectively and individually—where we have tried to understand the contemporary foundation upon which the current pronouncements about radiation risk are made, we have discovered the outlines of numerous debates and a context much larger than we anticipated.

In our last joint paper in 2015, we contrasted the perspectives of two geneticists, James V. Neel from the United States and Yuri Dubrova, originally from Ukraine but later a migrant to England. Our goal in that work was to try to understand the assertions of the Atomic Bomb Casualty Commission. In that work, too, while scientific uncertainty was central, we also considered the broader context of scientific training and other alliances that existed. James Neel’s status and scientific reputation, we wrote, “gave him the opportunity to help establish radiation safety standards for nuclear workers and the public. Through the interdependency of funding and prestige, they also enabled him and his colleagues to build the large-scale statistical models in population and epidemiological genetics that have become the gold standard for research, while other scientific methods and approaches were dismissed as substandard” (Goldstein and Stawkowski 2015, p. 92). In that essay, we take the construction of these quite durable findings from a different perspective: the research conducted among atomic bomb survivors since 1950 and the many subsequent reports addressing the risks associated with low dose ionizing radiation, which are renewed in every cycle of UNSCEAR work. The angle we take here has us approaching these epistemological questions differently, adhering to a specific focus on insect research. We hope that this alternative lens can help us further configure and better understand the endurance and forms of radiation risk knowledge and situate scientific movement over time.

One of the key contributions of this paper is to show that our understanding of genetic risks from radiation exposure has been directly shaped by the choice of model organisms—especially *Drosophila*—and the scientific networks that emerged in genetics. The dominance of *Drosophila* in genetics research, cemented through the work of Muller, Dobzhansky, and their students, created a particular way of thinking about mutation and the long-term population-level genetic effects of radiation exposure.^24^ We refer to this as the “*Drosophila* bias,” where alternative evidence of genetic harm, such as that found in radiation studies in Lepidoptera, has often been dismissed. Among these organisms, the fruit fly, *Drosophila melanogaster* (and others), occupies a central role in this production of knowledge. This tiny creature has played a critical role in our understanding of genetics, particularly in the early years of the discipline. Population biology and its surrounding disciplines are also key here. In the pre-DNA world *Drosophila* research in Brazil, the measurement of all things was supposed to lead to knowledge about populations—what they looked like, how they developed, what changes an irradiated laboratory fly versus a naturally occurring one might produce in that population—all to find out what the future populations of flies (and ultimately humans) would look like (see Goldstein 2017). It became common knowledge and even common sense during these early years, that insects could be exposed to a great deal of radiation and could survive, and that subsequent generations would not see too much destabilization. This helps to explain Muller’s simultaneous belief that irradiated flies were no more lethal than mutant flies in nature, a finding that seems contradictory to his lifelong quest to secure an understanding of the dangers of radiation. To say this in another way, Muller was able to adhere to the epistemic principles of genetics and population biology of the time, while still proposing that radiation could be harmful. Crow, however, found Muller’s worry about radiation to be overwrought, and the endless reports from UNSCEAR declaring that the risks to human populations are minimal—at least from our reading—hold to the core principles established by the original ABCC research directed by James V. Neel. While we are not entirely certain whether these principles are too stringent, what we do see is a tendency to downplay radiation concerns and envision a future built on a sense of resiliency in nature that would prevail for us all.

That is, until we get to Fukushima and the butterfly. In this case, we find an insect that ought to, theoretically, be able to handle the radiation effects of the nuclear meltdown, but instead, shows alarming signs of mutation and intergenerationally intensifying damage. In important ways, the studies of mutant butterflies from Fukushima provide an unanticipated counterpoint to the *Drosophila* insect model and call into question scientific assumptions about animal and human resilience to radiation exposure. The butterflies’ accumulation of damage across generations points to an alternative understanding of radiogenic harm, one that scientific reliance on *Drosophila* as the central model organism has overlooked. The research on butterflies not only revealed the presence of inherited genetic damage but also showed its exacerbation in second and third generations. Borrowing the concept of episteme from the historian of ideas and philosopher Michel Foucault, we consider the *Drosophila* bias of the early years of radiation risk work and wonder whether paying attention to Lepidoptera will move scientific research in these affected areas to consider the possibility of long-term genetic damage from nuclear accidents. We are uncertain what this shift could mean for the recovery of the butterfly or for that matter, for us humans. But we argue that these findings should give us pause and call into question current epistemological frameworks rooted in early to mid-20th century scientific traditions of insect research. In this context, model organisms should be understood as always biased tools of research: they shape what questions are asked, what evidence is considered, what biological/genetic risks are dismissed or recognized. Ultimately, what our analysis shows is that our current understanding of radiation exposure and biological/genetic effects is historically constructed and biased by the dominance of *Drosophila* as a model organism. This echoes Creager’s (2022) point that model organisms not only help define experimental approaches, but also shape broader regulatory frameworks, cementing certain assumptions into scientific practice in the process. Current radiation safety standards rest on epistemic foundations constructed through *Drosophila* research and Cold War era scientific priorities. But the Fukushima butterflies suggest something else: that cumulative, intergenerational genetic damage may indeed be possible in ways that our current framework is not prepared to recognize. Whether this applies to humans or not is still uncertain, but that uncertainty should promote scientific investigation rather than outright dismissal. It is in this spirit that we offer the Fukushima butterflies as an alternative to the fruit fly: this is a call for the re-examination not only of our scientific animal models, but also the assumptions about harm and resilience embedded within them.

## Data Availability

No datasets were generated or analysed during the current study.

## Footnotes

1 See Scientific Council on the Developing Child (2006, p. 1). While important parallels between animals and humans might, and often do, exist, the precise nature of such extrapolations remains a subject of uncertainty. Additionally, there is much debate about the distinct somatic (body cell-related, non-heritable) versus genetic (heritable, passed to offspring) effects of radiation exposure, a debate that stems from the uses of the first nuclear bombs in Hiroshima and Nagasaki. For instance, various chemicals with toxic properties are not only carcinogenic, but also affect brain development, heart and kidney function, and interact differently across species and among diverse individual bodies (Johnson et al. 2019; Tchounwou et al. 2012; Wing 1998) This intricate web of toxic effects and their pathways—where they might hide or accumulate to unknown effect—often extends to a combination of substances over time, a phenomenon known as “toxic layering” (Goldstein and Hall 2015).

2 We recognize that we compare different levels of taxonomic categories—humans (a species), *Drosophila* (a genus of fruit flies), and Lepidoptera (an order including butterflies and moths). Our use in this paper reflects how the historical actors themselves used these categories. For example, “*Drosophila*” often functioned as a shorthand for *Drosophila melanogaster* used in genetic research, while “Lepidoptera” referred to the diverse butterflies and moths studied in ecological and radiobiological contexts. The imprecise use of these categories is significant because it reveals how model organisms research created generalizable knowledge claims that crossed taxonomic boundaries (Bolman 2022; Creager 2022).

3 This was Goldstein’s field site in the 2000s.

4 Vilela, Carlos. Interview by Donna M. Goldstein. São Paulo, Brazil, May 22, 2012 and March 16, 2015. Perondini, André Luiz Paranhos.Interview by Donna M. Goldstein. São Paulo, Brazil, May 23, 2012.

5 Goldstein conducted oral history interviews (private collection) in 2012 and 2015 with several scholars who worked directly or indirectly with Dobzhansky in São Paulo. In kinship terms these were the same people, or the next generation down from the principal researchers who had studied with Dobzhansky in the 1940s and 1950s at the University of São Paulo.

6 Provine (1986) notes that Dobzhansky was relatively weak on methods and mathematical work, and relied on Sewall Wright for many of his innovations in this area.

7 The five conditions specified in the Hardy-Weinberg Equilibrium Principle are: (1) no mutation and no new alleles are generated by mutation, nor are genes duplicated or deleted; (2) random mating; (3) no gene flow; (4) very large population size; (5) no natural selection.

8 Vilela, Carlos. Interview by Donna M. Goldstein. São Paulo, Brazil, May 22, 2012 and March 16, 2015; Perondini, André Luiz Paranhos. Interview by Donna M. Goldstein. São Paulo, Brazil, May 23, 2012.

9 See also Goldstein (2012, 2013), Farish (2013), Immerwahr (2019).

10 Magalhães, Luiz Edmundo de. Interview by Donna M. Goldstein. São Paulo, Brazil, May 22, 2012.

11 Heterosis refers to the phenomenon in progeny of diverse varieties of a species, or crosses between species who exhibit greater biomass, speed of development, and fertility than both parents. Various models have been posited to explain heterosis, including dominance, overdominance, and pseudo-overdominance.

12 See Beatty (1987) for a detailed discussion of this controversy. Comfort (2012) notes this ironic naming of “classical” by Dobzhansky to connote what Dobzhansky considered the stuffiness and obsolescence of Muller’s position.

13 See Crow (1987) for more on this. Crow’s role in understanding the post-War ABCC reports is crucial. He headed a committee in 1975 to review the original report.

14 See also Pavan and Brito da Cunha (2003), Toledo et al. (1966).

15 In the 1980s, for example, Rosalie Bertell interpreted Muller’s *Drosophila* research as predicting a reduction in the survival ability of the human species as several generations are exposed to ionizing radiation, a prediction that Bertell assumes was buried—or kept secret—by the Atomic Energy Commission in its consistent mission to underplay the harms caused by atomic weaponry and atomic energy (Bertell 1986).

16 Indeed, this very same journal, *Journal of the History of Biology*, devoted most of an issue (Vol. 20, No. 3, Autumn 1987) to the Muller-Dobzhansky debate that we have depended on for building our perspective, most significantly papers by Crow (1987) and Beatty (1987). More recently, the same journal once again revisited questions of experimental organisms in a special issue (Vol. 55, No. 1, 2022) focusing on the ethical and political entanglements of model organism studies, in particular, essays by Bolman (2022), Kohler (2022), Creager (2022), and Rader (2022).

17 Insects in general, including *Drosophila*, are known to have great resistance to radiation. Adult insects are at least 100 times less sensitive to the lethal effects of radiation than are vertebrates, and mammals are more radiosensitive than birds, fish, amphibians, or reptiles (O’Brien and Wolfe 1964). This scientific understanding of radiosensitivity among different animals has remained mostly unchanged, with more recent reviews confirming that insects (invertebrates) are generally the most radioresistant group of animals, even if radiation resistance varies to some degree across insect orders ( Lourenço et al. 2023; Paithankar et al. 2022).

18 In fact, unlike research on the Sellafield and other nuclear installations in England, where discussions have centered on the link between childhood leukemia and parental radiation exposure, Fukushima’s scientific discourse has mostly ignored genetic concerns (Kendall et al. 2016; and see Liu et al. 2022). Yet many residents and some scientists dispute the official narrative, arguing instead that long-term monitoring is essential for detecting genetic consequences many years down the road (Polleri 2019, 2026).

19 See for example, Lindee (1994) and Goldstein and Stawkowski (2015).

20 Mousseau, Timothy. Interview by Magdalena E. Stawkowski. Columbia, South Carolina, April 21, 2024.

21 Mousseau, Timothy. Interview by Magdalena E. Stawkowski. Columbia, South Carolina, April 21, 2024.

22 Mousseau, Timothy. Interview by Magdalena E. Stawkowski. Columbia, South Carolina, November 3, 2023.

23 See also UNSCEAR (2015) and UNSCEAR (2016).

24 Smocovitis (2012) points out that Dobzhansky ought to be credited, among other things, as the intellectual force that extended the modern evolutionary synthesis to include humans and human evolution. Indeed, Dobzhansky’s work made it possible for anthropology to become part of the debates in evolutionary biology.
